# Dynamic changes in microbial communities during the bioremediation of herbicide (chlorimuron-ethyl and atrazine) contaminated soils by combined degrading bacteria

**DOI:** 10.1371/journal.pone.0194753

**Published:** 2018-04-05

**Authors:** Jian Wang, Xinyu Li, Xu Li, Huanhuan Wang, Zhencheng Su, Xiujuan Wang, Huiwen Zhang

**Affiliations:** 1 Key Laboratory of Pollution Ecology and Environmental Engineering, Institute of Applied Ecology, Chinese Academy of Sciences, Shenyang, China; 2 University of Chinese Academy of Sciences, Beijing, China; Babasaheb Bhimrao Ambedkar University, INDIA

## Abstract

Chlorimuron-ethyl and atrazine are two herbicides with long half-lives in soil; their long-term and excessive application has led to a series of environmental problems. In this study, the strains *Chenggangzhangella methanolivorans* CHL1 and *Arthrobacter* sp. ART1 were combined and used for the remediation of chlorimuron-ethyl, atrazine and combined contaminated soils in a microcosm experiment. Changes in chlorimuron-ethyl and atrazine concentrations in soils were monitored, and variations in the soil microbial community were studied by phospholipid fatty acid (PLFA) analysis. The two inoculated degrading strains accelerated the degradation of chlorimuron-ethyl and atrazine in soil, especially in the combined contaminated soil. Addition of the two herbicides and their combination generally decreased the concentrations of total PLFAs, total bacterial PLFAs, Gram-negative and Gram-positive bacterial PLFAs and Shannon-Wiener indices, and changed microbial community composition, whilst stimulating fungal PLFA concentrations. In addition, the combined herbicide treatment had more impact on microbial biomass than the single herbicide treatments. Inoculation treatments significantly relieved the effects of herbicides on soil microbial biomass, diversity and community structure. This study demonstrated that strains CHL1 and ATR1 have the potential to remediate chlorimuron-ethyl, atrazine and combined contaminated soils, and provided valuable information for remediation of chlorimuron-ethyl, atrazine and combined contaminated soils *in situ*.

## Introduction

Herbicides have been widely used all over the world for controlling weeds and have made a great contribution to modern agriculture. Chlorimuron-ethyl and atrazine were used for pre- and postemergence broadleaf weed control for soybean and maize crops, respectively; they are characterized by very low application rates, high herbicidal activity and good crop selectivity [1–3]. However, the long-term and excessive application of chlorimuron-ethyl and atrazine could result in high concentrations of soil residues. In the north of China, combined soil pollution with chlorimuron-ethyl and atrazine residues often occurs because of the rotation of maize and soybean.

Soil chlorimuron-ethyl and atrazine residual pollution could significantly shift the soil microbial community structure and function, and affected the growth of sensitive succeeding crops even at low concentrations [4, 5]. In addition, chlorimuron-ethyl and atrazine are considered potentially hazardous pollutants to human health [6, 7]. The chlorimuron-ethyl is absorbed from the gastrointestinal tract and is distributed throughout the body, with the largest portions found in the liver[8]. Long-term exposure to atrazine causes breast and ovarian cancer. It may also cause circulatory disturbance in poisoned animals. The countries such as United States, Japan and the European Union have placed atrazine on the list of endocrine disruptors [9–11]. Therefore, the remediation of chlorimuron-ethyl and atrazine contaminated soil is increasingly necessary and urgent.

Generally, the environmental fate of chlorimuron-ethyl and atrazine are mainly chemical degradation and biodegradation, among which chemical degradation includes photolysis and hydrolysis. Chlorimuron-ethyl and atrazine degradations in soil mainly occur by hydrolysis and biodegradation. The pH of the aqueous solution and soil is one of the influencing factors for the hydrolysis of chlorimuron-ethyl and atrazine.

Chlorimuron-ethyl is stable in neutral and weak alkaline aqueous solution (pH = 7.0–9.0), but hydrolyzes more quickly in acidic buffer solution (pH ≤ 4.0) [12]. The half-life is 14 days in water (pH = 4). Chlorimuron-ethyl is less volatile and its half-life in soil is approximately 7–70 days depending upon soil characteristics [13]. The persistence of chlorimuron-ethyl increases with increasing pH. Atrazine is almost non-volatile and its half-life in soil, water, and sediments ranges from few weeks to more than 2 years depending on various environmental factors like pH, moisture content, temperature and soil humus and clay minerals [14–17]. Hydrolysis of atrazine is rapid under acidic or basic conditions but is slower at neutral pHs[18]. In addition, atrazine is extremely persistent in clay and sandy loam soils. In addition, atrazine is extremely persistent in clay and sandy loam soils[19, 20].

Microbial transformation also has an important role on degradations of chlorimuron-ethyl and atrazine. Many microbes that can degrade chlorimuron-ethyl or atrazine have been isolated and characterized, but the degradation ability of most of these microbes was assessed under laboratory culture conditions [21, 22]. A few studies have focused on soil bioremediation using degrading microorganisms in single herbicide contaminated soil [23]. However, few studies have investigated the bioremediation of combined herbicide contamination.

In this study, we combined the highly efficient chlorimuron-ethyl degrading strain *Chenggangzhangella methanolivorans* CHL1 [24] and the highly efficient atrazine degrading strain *Arthrobacter* sp. ATR1 to remediate chlorimuron-ethyl and atrazine contaminated soils. To assess the potential for bioremediation of chlorimuron-ethyl and atrazine contaminated soils by combined degrading bacteria, chlorimuron-ethyl and atrazine residues and dynamic variations in the microbial community during the remediation process were investigated. This study will contribute to improving the remediation of combined pesticide pollution.

## Materials and methods

### Soil sampling

Soil samples (brown loam; 0–15 cm in depth) were obtained from abandoned land that had not previously had herbicides and fertilizers applied for 16 years at the National Field Research Station of Shenyang Agroecosystems, Shenyang, China (41°47'N, 123°23'E). The soil was mixed and sieved through a 2-mm mesh, and stored at room temperature until experimental treatments were commenced the next day. The physical and chemical properties of soil were as follows: 13.9 g kg^−1^ organic matter, 25.9 mg kg^−1^ available nitrogen, 11.2 mg kg^−1^ available phosphorus, and 135.7 mg kg^−1^ available potassium, with a pH of 6.62.

### Chemicals and media

Chlorimuron-ethyl (purity 98.70%), atrazine (purity 97.50%) and reagents used for chromatographic and spectroscopic analysis were all purchased from Sigma-Aldrich Chemical Co. (Shanghai, China). All other chemicals and solvents were of analytical grade. Strains *Chenggangzhangella methanolivorans* CHL1 and *Arthrobacter* sp. ATR1 were previously isolated and stored in our laboratory. Mineral salt medium (MSM) contained 2.0 g NaNO_3_, 2.0 g KH_2_PO_4_, 0.5 g NaCl, 0.125 g MgSO_4_∙7H_2_O, 0.02 g FeSO_4_∙7H_2_O, 0.2 g yeast extract, and 10 mL methanol in 1000 mL distilled water, pH 7.0. Nutrient broth (NB) medium contained 10 g peptone, 5 g beef extract, and 5 g NaCl in 1000 mL distilled water, pH 7.0.

### Experimental design and treatments

Sieved soil was thoroughly homogenized to eliminate possible heterogeneity between replicates. The soil was distributed into eight groups and treated separately as described in Table 1. For each treatment, 3 kg of soil was put in a pot (diameter, 18 cm; depth, 25 cm) with three replicates.

**Table 1:** Experimental treatments used in this study.

Treatment Code	Chlorimuron-ethyl (μg kg^-1^)	Atrazine(mg kg-1)	Combined degrading bacteria
**CK-**	0	0	uninoculated
**CK_+_**	0	0	inoculated
**CHL-**	500	0	uninoculated
**CHL_+_**	500	0	inoculated
**ATR-**	0	10	uninoculated
**ATR_+_**	0	10	inoculated
**CHL+ATR-**	500	10	uninoculated
**CHL+ATR_+_**	500	10	inoculated

“CK” indicates control treatment group, “CHL” indicates chlorimuron-ethyl treatment group, “ATR” indicates atrazine treatment group, “CHL+ATR” indicates chlorimuron-ethyl and atrazine treatment group, “-” indicates without bacterial consortium inoculation, “+” indicates with bacterial consortium inoculation.

Strain CHL1 was cultured in MSM liquid medium and ATR1 was cultured in NB liquid medium until they reached the stationary phase. Cell pellets of the two strains were collected and washed twice with sterile phosphate buffered saline (PBS). Finally, the cell pellets of CHL1 and ATR1 were suspended in PBS and adjusted to 1.06×10^8^ and 1.4×10^8^ CFU g^−1^, respectively. The ratio of strains CHL1 and ATR1 in the mixture was 1:1 (v:v). Sterile deionized water was added to adjust the soil initial moisture content to 40%. The pots were incubated for 60 days under outdoor natural conditions (25–28°C). Soil samples were periodically removed for chlorimuron-ethyl and atrazine residue determination and PLFA analysis on days 1, 7, 14, 30, 45 and 60.

### Determination of residual chlorimuron-ethyl and atrazine in soils

Soil samples (20 g) were weighed into 250 mL Erlenmeyer flasks to which 25 mL 0.1% aqueous ammonia solution and 25 mL methanol were added. After sonication extraction for 20 min, the mixture was centrifuged (4000×g, 5 min). The extraction was repeated three times, and the supernatants were merged. A Cleanert HXN cartridge (500 mg 6 mL^−1^, Agela Technologies Inc.) was used to purify the residue. The elution was dried under N_2_, resuspended in 1 mL methanol and filtered through a 0.22 μm nylon filter. The concentrations of chlorimuron-ethyl and atrazine in soils were analyzed by HPLC equipped with a Zorbax C-18 ODS Spherex column (4.6 × 250 mm, 5 μm, Agilent Technologies, Palo Alto, CA, USA). Detection of chlorimuron-ethyl and atrazine was performed at 254 nm and 220 nm, respectively, with a mobile phase consisting of 0.5% acetic acid:methanol (30:70, v/v) at a flow rate of 1 mL min^−1^ [25, 26]. The injection volume was 10 μL of each solution for detection.

### Analysis of soil microbial community structure

PLFA analysis have been employed to study the effects of herbicides on microbial abundance, community structure, nutritional and physiological status [27, 28]. Soil lipids extraction was performed according to the procedure described [29]. Briefly, lyophilized soil samples (4 g) were extracted with Bligh-Dyer solution (chloroform: methanol: citrate buffer (pH = 4), 1:2:0.8; v/v/v) in Teflon screw cap culture tubes. The extraction was repeated twice. The chloroform layer was then collected and dried under N_2_. The total lipid extract was fractionated into neutral lipids, glycolipids, and polar lipids by a Cleanert Silica cartridge (500 mg 6 mL^−1^, Agela Technologies Inc.) The polar lipids were transesterified into fatty acid methyl esters (FAMEs) by methylation. The final solution of FAMEs was analyzed by GC using the Sherlock system (MIDI Inc., Newark, DE, USA). The quantifications of fatty acids were based on the 19:0 internal standard peak areas.

Samples were injected at a split ratio of 30:1 and fatty acids from 10 to 24 carbon atoms were identified. Fatty acids used as markers for bacteria were i14:0, i15:0, 15:0, a15:0, i16:0, 16:1ω7, i17:0, a17:0, cy17:0, 17:0 and cy19:0 [29, 30]. The branched phospholipids i14:0, i15:0, a15:0, i16:0, i17:0 and a17:0 were used as markers for Gram-positive (GP) bacteria, cy17:0, cy19:0, 17:1ω9c, 16:1ω7c, and 18:1ω7c for Gram-negative (GN) bacteria [30, 31]. Fatty acids 18:2ω6 and 18:1ω9c were employed as fungal markers [32–34]. The total amount of PLFA was calculated to indicate the total microbial biomass.

### Data analysis and statistics

All values reported in this paper are the mean (± standard deviation) of three replicates. Total PLFA content, B/F and GN/GP ratio and Shannon index were subjected to two-way or three-way analysis of variance (ANOVA) by SPSS 19.0 (SPSS, Chicago, Illinois, USA). The least significant difference (LSD) test was used to separate means at P <0.05. Principal components analysis (PCA) was performed using R software.

## Results

### Chlorimuron-ethyl and atrazine degradation in experimental soils

The concentrations of chlorimuron-ethyl and atrazine during the incubation period in experimental soils are shown in Table 2. Both chlorimuron-ethyl and atrazine concentrations in herbicide contaminated treatments gradually decreased with incubation time. However, the degradation rates of both chlorimuron-ethyl and atrazine were higher in inoculated treatments than in uninoculated treatments. From day 7 onwards, the concentrations of the two herbicides were significantly lower in inoculated treatments than in uninoculated treatments. After 45 days, the chlorimuron-ethyl residues in inoculated treatments were below the minimum detectable level, but in uninoculated treatments, chlorimuron-ethyl residues were more than 10 μg kg^−1^ at the end of the incubation period. The concentrations of atrazine in inoculated and uninoculated treatments were below the minimum detectable level on days 30 and 45, respectively. The degradation rates of both chlorimuron-ethyl and atrazine were higher in the combined herbicide treatment than in the single herbicide treatments. For inoculated and uninoculated treatments, the residual concentrations of the two herbicides were significantly lower in the combined herbicide treatment than in the single herbicide treatments from days 7 and 14 onwards, respectively.

**Table 2:** Chlorimuron-ethyl and atrazine concentrations in different treatments over time.

Herbicide	Treatments	0d	7d	14d	30d	45d	60d
**Chlorimuron-ethyl (μg/Kg)**	*CHL_-_	480.89a	250.16b	168.36a	149.27a	89.61a	18.27a
	CHL_+_	479.77a	20.59c	18.57c	7.23b	N	N
	CHL+ATR_-_	500.49a	330.11a	117.00b	85.1c	62.53b	10.8b
	CHL+ATR_+_	490.11a	9.2d	4.7d	4.1d	N	N
**Atrazine** **(mg/Kg)**	ATR_-_	9.33a	5.03a	2.14a	0.079a	N	N
	ATR_+_	9.46a	3.04b	0.92b	N	N	N
	CHL+ATR_-_	9.48a	5.62a	1.13c	0.071a	N	N
	CHL+ATR_+_	9.17a	3.02b	0.24b	N	N	N

Values represent the mean of three replicates.

* “CHL” indicates chlorimuron-ethyl treatment group, “ATR” indicates atrazine treatment group, “CHL+ATR” indicates chlorimuron-ethyl and atrazine treatment group.

“-” indicates uninoculated treatments

“+” indicates inoculated treatment.

“N” indicates that the chlorimuron-ethyl or atrazine was not detected. Different lower-case letters indicate significant differences between concentration of herbicide in each column at *P* <0.05.

### Changes in PLFA concentrations in experimental soils

**Fig 1:**
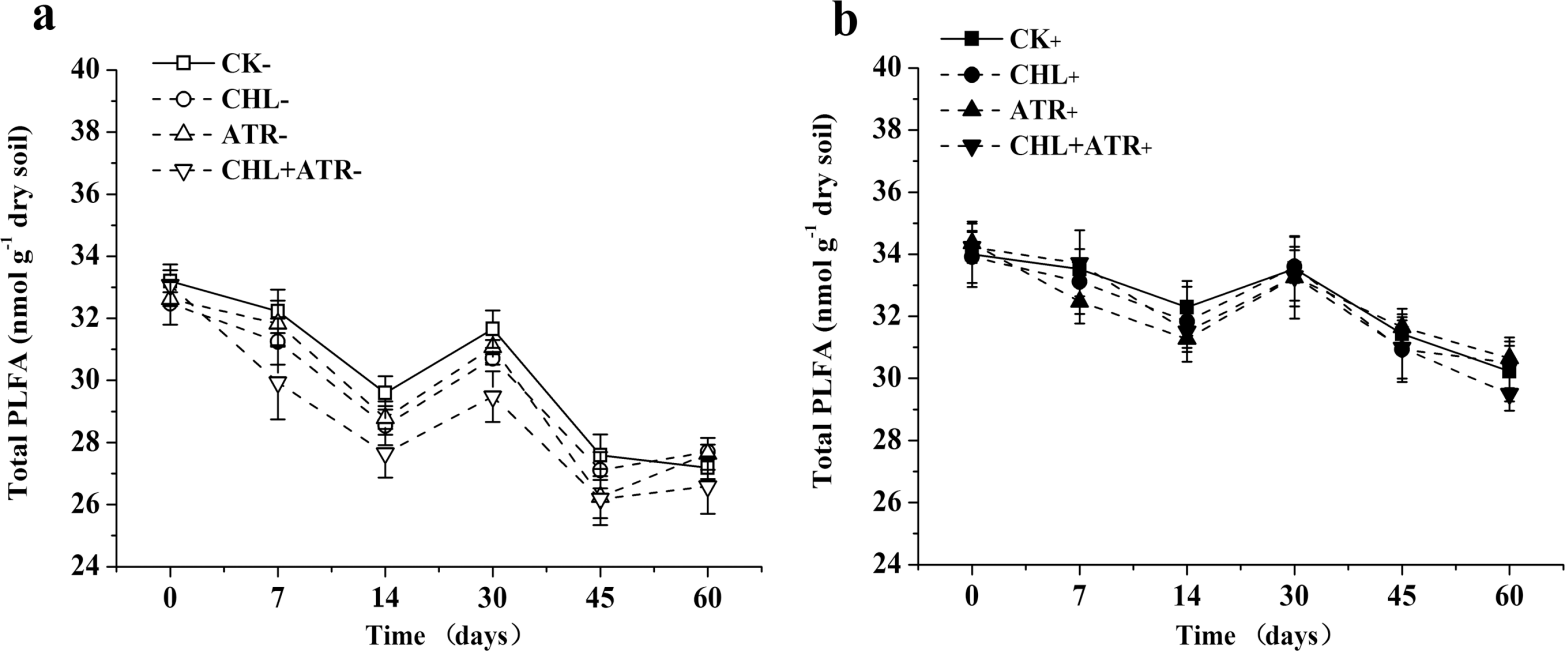
Total phospholipid fatty acid (PLFA) biomass in inoculated (a) and uninoculated (b) treatments. Symbols represent the mean of triplicate samples and error bars indicate the standard deviation. “CK” indicates control treatment group, “CHL” indicates chlorimuron-ethyl treatment group, “ATR” indicates atrazine treatment group, “CHL+ATR” indicates chlorimuron-ethyl and atrazine treatment group, “-” indicates uninoculated treatments, “+” indicates inoculated treatments.

The total PLFA concentrations of experimental soils ranged from 26.25 to 33.20 nmol g^−1^ in uninoculated treatments, and from 29.45 to 34.35 nmol g^−1^ in inoculated treatments (Fig 1). The total PLFA concentrations were significantly higher in inoculated treatments than in uninoculated treatments throughout the incubation period (F_Time_ = 59.625, p<0.001) (Table 3). The single and combined herbicide treatments all had an inhibitory effect on total PLFA concentrations, with greater inhibition for the combined herbicide treatment than for the single herbicide treatments (Fig 1). In uninoculated treatments, the combined herbicide treatment significantly decreased total PLFA concentrations when compared with the control uninoculated treatment (CK_−_) on days 7 and 30 (Fig 1A). In the inoculated treatments, the total PLFA concentrations were not significantly different among all treatments from day 7 onwards (Fig 1B). The decrease in total PLFA concentrations was lower in herbicide treatments with inoculation than without inoculation throughout the incubation period when compared with CK_+_ and CK_−_ treatments, respectively. After 30 days, the total PLFA concentrations gradually recovered to the control level in uninoculated herbicide treatments.

**Table 3:** Multivariate analysis of variance by three-way ANOVA of the sum of all PLFAs, Gram-negative bacteria(GN), Gram-positive bacteria(GP), bacteria, fungi, and the Shannon index.

^a^Factor	Total PLFA	GN	GP	Bacteria	Fungi	Shannon index
**Treatment(treat)**	18.120***	13.014***	13.360***	26.858***	15.403***	18.619***
**Inoculation(In)**	13.803*	20.115***	19.896***	9.008***	8.491***	33.897***
**Time**	59.625***	20.783***	56.960***	76.218***	19.987***	18.512***
**Treat×time**	18.781***	24.035***	14.557***	16.766***	14.793***	26.007***
**In×treat**	14.693**	13.689**	16.198***	18.048***	42.650***	17.369***
**In×time**	20.951**	30.408***	31.228***	41.085***	11.185***	33.052*
**In×treat×time**	34.106***	28.875**	15.614*	23.536***	32.037**	23.709***

^a^The categorical factors are treatment (CK: control soil; CHL: chlorimuron-ethyl soil; ATR: atrazine soil; CHL+ATR: chlorimuron-ethyl and atrazine soil), inoculation (uninoculated treatments, inoculated treatments) and incubation time (1, 7, 14, 30, 45 and 60 days). Presented are the *F*-values with the level of significance (p<0.05*, p<0.01** and p<0.001***).

### Changes in bacterial and fungal PLFAs in experimental soils

**Fig 2:**
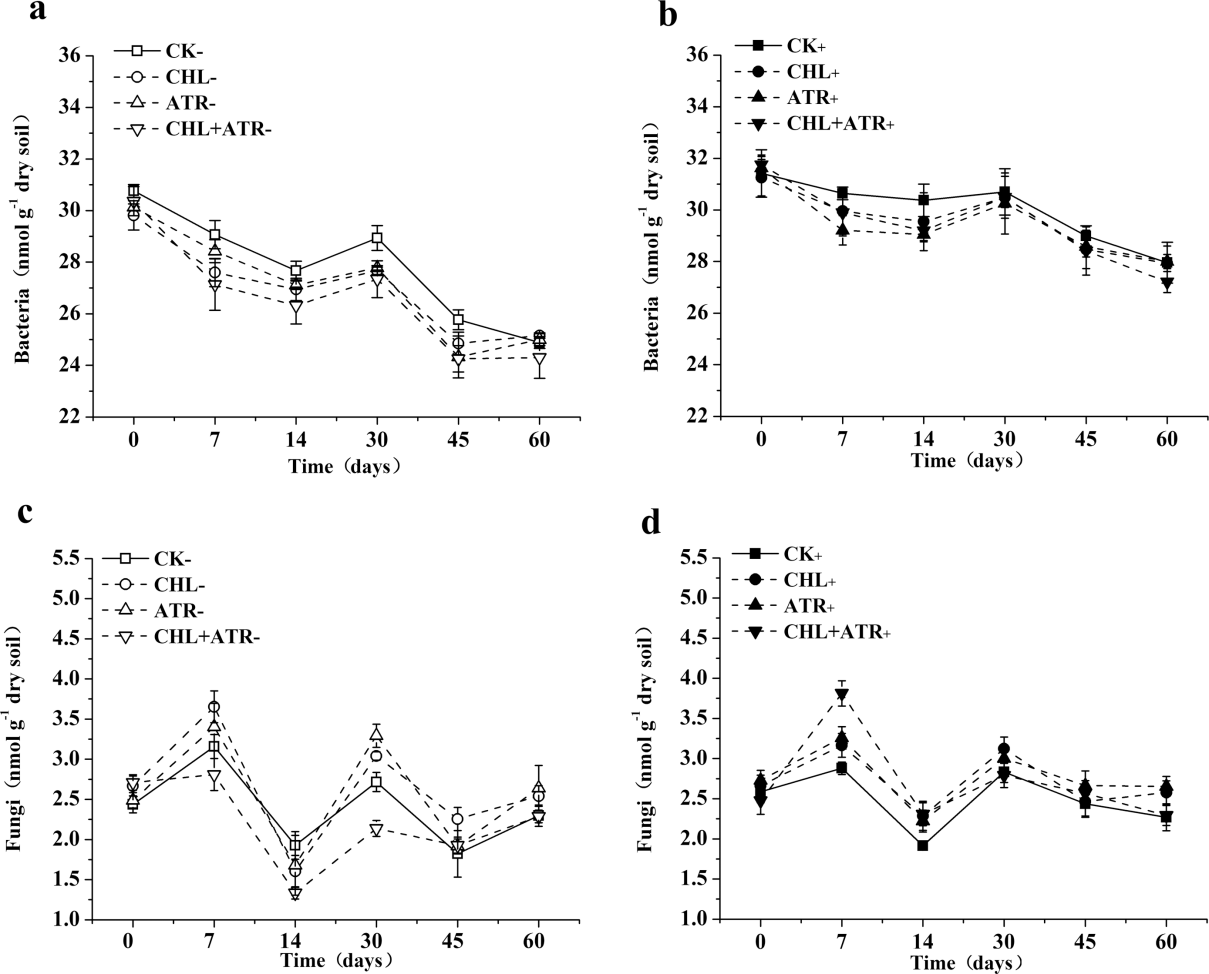
Bacterial and fungal abundance in uninoculated (a, c) and inoculated (b, d) treatments. Symbols represent the mean of triplicate samples and error bars indicate the standard deviation. “CK” indicates control treatment group, “CHL” indicates chlorimuron-ethyl treatment group, “ATR” indicates atrazine treatment group, “CHL+ATR” indicates chlorimuron-ethyl and atrazine treatment group, “-” indicates uninoculated treatments, “+” indicates inoculated treatments.

All herbicide treatments showed persistent inhibition of soil bacteria throughout the incubation period, and the combined herbicide treatment showed more inhibition than either of the single herbicide treatments (Fig 2). On days 7 and 30, all uninoculated herbicide treatments had significantly lower bacterial PLFA concentrations when compared with CK_−_ (Fig 2A). For soil fungi, both the single chlorimuron-ethyl and atrazine treatments generally exhibited positive effects on fungal PLFA abundance, however there was a negative effect on soil fungal PLFAs in the combined treatment without inoculation (Fig 2C). On days 7 and 30, the single uninoculated herbicide treatments showed significantly increased fungal PLFA concentrations when compared with CK_−_, whereas the combined herbicide treatment resulted in significantly decreased fungal PLFA concentrations (Fig 2C). The effects of herbicide treatments on bacteria and fungi were significantly relieved by the inoculation treatments (F_Inoculation_ = 8.491, p<0.001) (Fig 2B and 2D, Table 3). Although the PLFA concentrations of bacteria and fungi in the uninoculated treatments recovered by the end of the incubation, inoculation accelerated the recovery process. For inoculated and uninoculated treatments, there was no obvious difference between the herbicide treatments and the control from days 30 and 45 onwards, respectively. In addition, the inoculated herbicide treatments showed lower inhibition of the bacterial PLFA concentrations than the uninoculated herbicide treatments when compared with their corresponding controls. Further, inoculation reduced the stimulatory effect on soil fungi in the single chlorimuron-ethyl and atrazine treatments from day 14 onwards, and relieved the inhibitory effect on soil fungi in the combined herbicide treatment.

### Changes in GN and GP PLFAs in experimental soils

**Fig 3:**
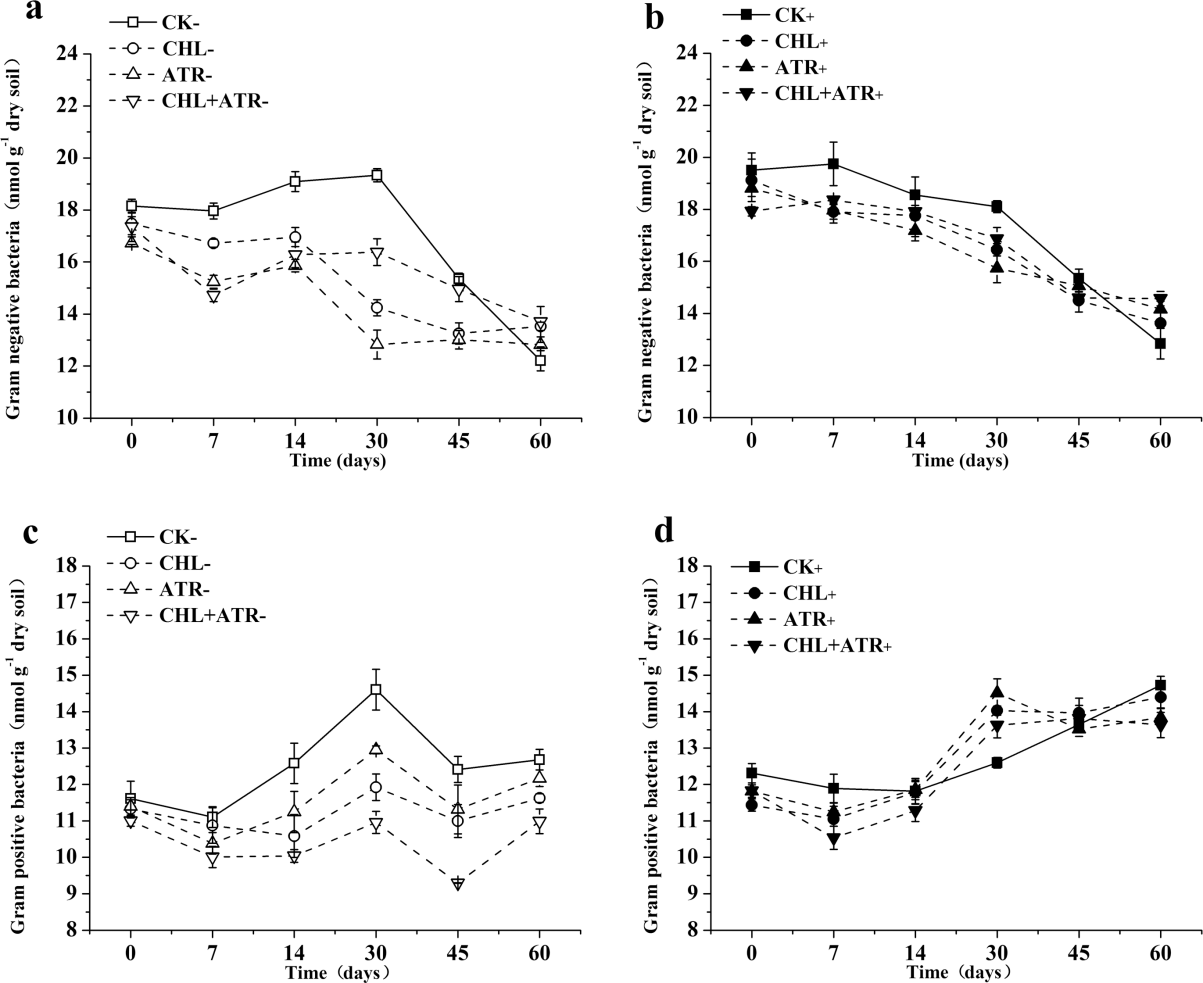
Gram-negative (GN) and Gram-positive (GP) abundances in uninoculated (a, c) and inoculated (b, d) treatments. Symbols represent the mean of triplicate samples and error bars indicate the standard deviation. “CK” indicates control treatment group, “CHL” indicates chlorimuron-ethyl treatment group, “ATR” indicates atrazine treatment group, “CHL+ATR” indicates chlorimuron-ethyl and atrazine treatment group, “-” indicates uninoculated treatments, “+” indicates inoculated treatments.

All herbicide treatments decreased the PLFA concentrations of GN and GP bacteria (Fig 3). In uninoculated treatments, the PLFA concentrations of GN and GP bacteria in herbicide treatments significantly decreased from 7 to 30 days and from 14 to 45 days, respectively, when compared with those in CK_−_ (Fig 3A and 3C). The inhibitory effect of herbicides on PLFA concentrations of GN and GP bacteria decreased in the inoculated treatments, and the decrease in PLFA concentrations of GN and GP bacteria was lower in inoculated herbicide treatments than in uninoculated herbicide treatments when compared with those in CK_+_ and CK_−_ treatments, respectively (Fig 3B and 3D). On day 60, the PLFA concentrations of GN and GP bacteria in uninoculated treatments returned to the control level.

### Changes in Shannon-Wiener indices in experimental soils

**Fig 4:**
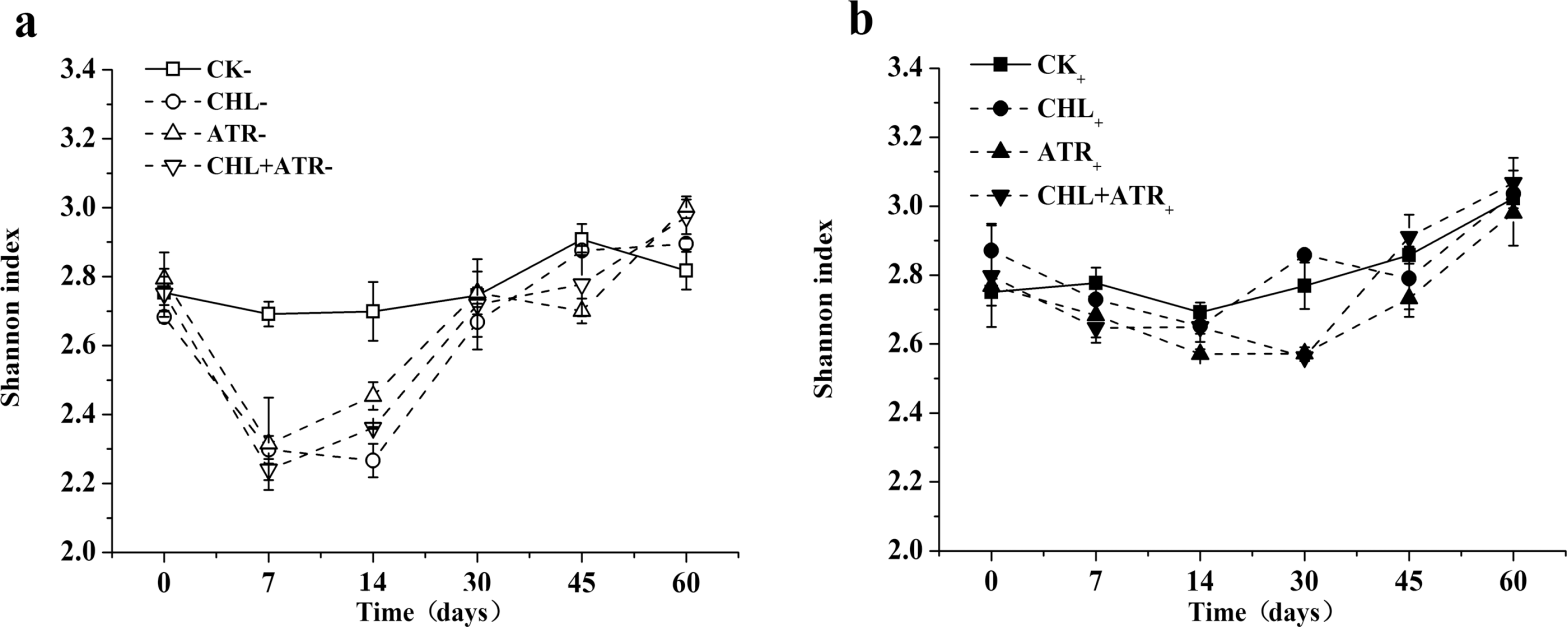
The Shannon-wiener index under inoculated treatments (a) and uninoculated (b) treatments in experimental soils. Symbols represent the mean of triplicate samples and error bars indicate the standard deviation. “CK” indicates control treatment group, “CHL” indicates chlorimuron-ethyl treatment group, “ATR” indicates atrazine treatment group, “CHL+ATR” indicates chlorimuron-ethyl and atrazine treatment group, “-” indicates uninoculated treatments, “+” indicates inoculated treatments.

Herbicide treatments significantly affected the Shannon-Wiener diversity index (F_Herbicide_ = 8.619, p<0.001). In uninoculated treatments, chlorimuron-ethyl, atrazine and their combination all significantly decreased soil microbial diversity on days 7 and 14 (Fig 4A). Inoculation of strains CHL1 and ATR1 significantly increased soil microbial diversity (F_Inocubation_ = 33.897, p<0.001) (Fig 4B, Table 3). In inoculated herbicide treatments, the Shannon-Wiener index almost recovered to the same level as in CK_+_ from day 7 onwards.

### Microbial community structures in experimental soils

**Fig 5:**
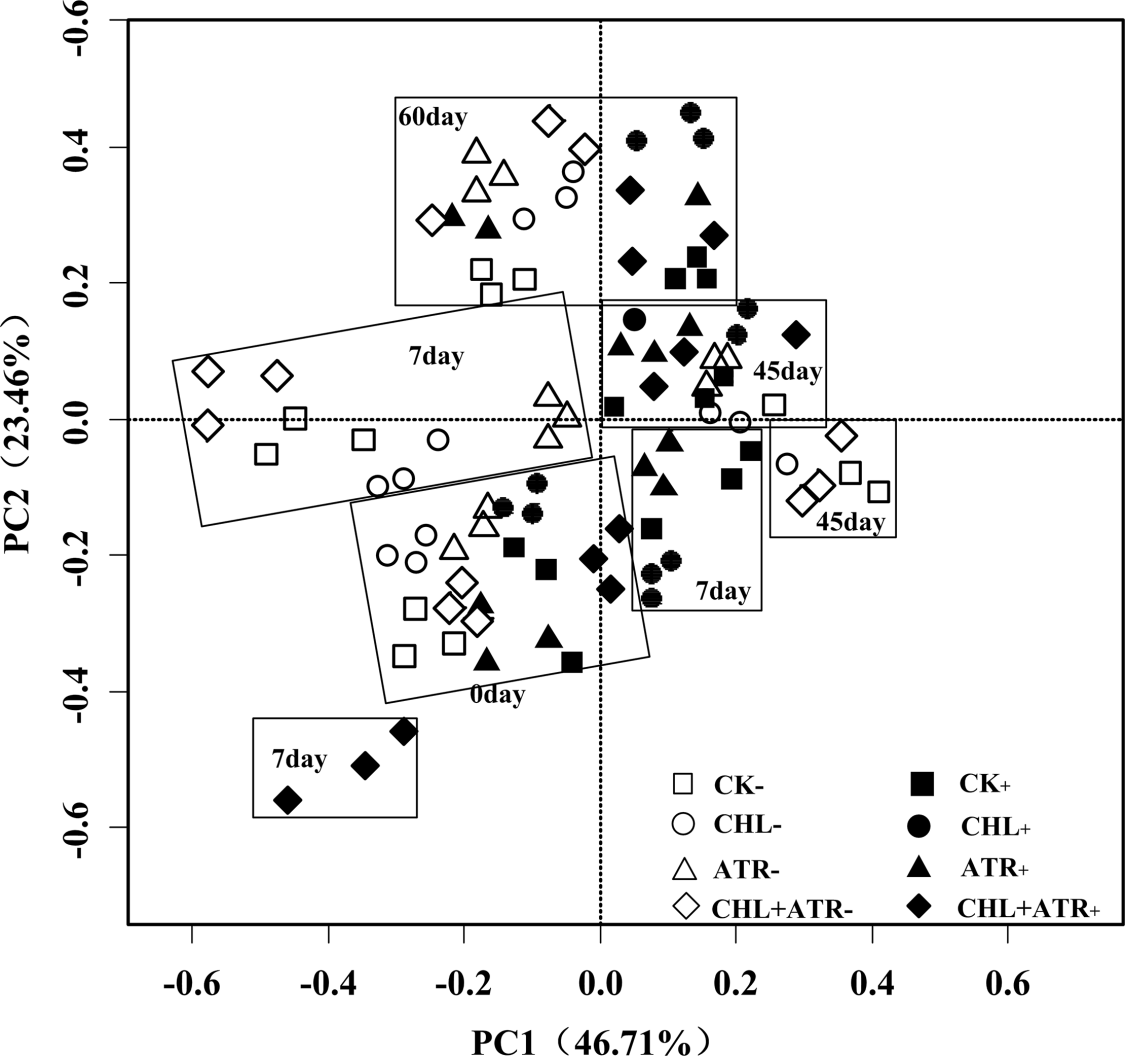
Principal component analysis plot of the microbial community structure of all treatments on days 0, 7, 45 and 60. “CHL” indicates chlorimuron-ethyl treatment group, “ATR” indicates atrazine treatment group, “CHL+ATR” indicates chlorimuron-ethyl and atrazine treatment group, “-” indicates uninoculated treatments, “+” indicates inoculated treatments.

The results of the principal component analysis (PCA) of PLFAs is presented in Fig 5. The first two principal component (PCs) accounted for 46.71% and 23.46%, respectively, of the variation in microbial community structure. Herbicide treatment, inoculation treatment and incubation time all affected the microbial community composition. All treatments clustered into different groups according to incubation time (0, 7, 45 and 60 days). In addition, the inoculated treatments were separated from the uninoculated treatments on day 7, and the inoculated treatments clustered together more closely than the uninoculated treatments except for the combined herbicide treatment with inoculation. On days 45 and 60, all treatments clustered closely together, which was the same as that observed on day 0. These results suggested that the two herbicides and their combination had a relatively large effect on the microbial community composition on day 7, and the inoculation of the degrading strains reduced these effects.

## Discussion

### Chlorimuron-ethyl and atrazine degradation in experimental soils

Bioremediation has been proven to be an effective alternative to costly traditional physicochemical techniques [35]. A series of bacterial strains were isolated from soil and were reported to have potential for the bioremediations of chlorimuron-ethyl and atrazine contaminated soil. Silvahttp://xueshu.baidu.com/s?wd=author%3A%28Elisabete%20Silva%29%20&tn=SE_baiduxueshu_c1gjeupa&ie=utf-8&sc_f_para=sc_hilight%3Dperson et al.(2004) described the use of strain *Pseudomonas* sp. ADP for bioremediation of atrazine-contaminated soils, where it degraded 30% of 168.7 mg/kg atrazine during incubation for 7 days [36]. Xiong et al. (2013) isolated the bacterial strain *Rhodococcus* sp. D310-1, which degraded 45% of 1000 mg L^−1^ chlorimuron-ethyl in contaminated soil during incubation for 45 days [37]. However, most isolated strains have been used to bioremediate soil contaminated with a single herbicide, with few examples of the remediation of combined herbicide contaminated soil. In this study, strains CHL1 and ATR1 were used to remediate atrazine, chlorimuron-ethyl and combined contaminated soils. Strains CHL1 and ATR1 were effective in degrading chlorimuron-ethyl and atrazine, and increased the degradation rate of atrazine and chlorimuron-ethyl in atrazine, chlorimuron-ethyl and combined contaminated soils. In addition, the degradation rates of both chlorimuron-ethyl and atrazine in the combined herbicide treatment were higher than in the single herbicide treatments. The results demonstrated that strains CHL1 and ATR1 showed promise for the remediation of atrazine, chlorimuron-ethyl and combined herbicide contaminated soil.

### PLFA concentration

The total amount of phospholipids is a good indicator of the living microbial biomass in soil [38, 39]. Previous researchers had reported that residual contamination with chlorimuron-ethyl and atrazine in cropland had negative effects on the soil microbes [24, 40]. Because of the chemical hydrolysis and degradation by indigenous microorganisms, atrazine and chlorimuron-ethyl contaminated soil might display intrinsic remediation within a certain range of concentrations [41, 42]. However, this process could be slow, and microbes that are sensitive to the two herbicides might be severely inhibited during this period. In this study, the inhibitory effects of herbicide contamination on total PLFA concentrations were relieved by the inoculation treatments. The PLFA concentrations returned to approximately the same levels as in the control within a shorter time period with inoculation than without, which is important for maintaining the sustainable development and ecological balance of soil ecosystems during bioremediation [43].

Herbicides can alter the bacterial/fungal ratio, often resulting in an increase in fungal biomass [44]. Yang et al. (2015) and Banks et al. (2014) found that chlorimuron-ethyl and atrazine, respectively, reduced bacterial biomass in soil [40, 45]. The results of this study were in accordance with previous studies; the bacterial PLFA concentrations were inhibited, but fungal PLFA concentrations were stimulated by chlorimuron-ethyl and atrazine in uninoculated treatments. However, the effects of herbicides on soil bacterial and fungal PLFA concentrations were significantly reduced in the inoculated treatments, which demonstrated that CHL1 and ATR1 are beneficial in restoring the soil microbial biomass balance in herbicide contaminated soils.

Owing to their different cell membrane structures, GN bacteria are more susceptible to environmental changes than GP bacteria, and GP bacteria are considered to be tolerant to herbicide stress [46–48]. Similar results were observed in this study. In uninoculated treatments, chlorimuron-ethyl and atrazine showed significant inhibitory effects on GN and GP bacteria on days 7 and 14, respectively. In inoculated treatments, the abundance of GP bacteria increased with incubation time, and the ratio of GN/GP decreased. This phenomenon was particularly pronounced in the combined herbicide contaminated soils. When compared with uninoculated treatments, inoculation with strains CHL1 and ATR1 decreased the variation in GN and GP abundance in soils.

The Shannon-Wiener index has often been used to study variations in microbial community diversity in herbicide contaminated soil [49]. Chlorimuron-ethyl and atrazine were reported to decreased the diversity of the soil microbial community [32, 50]and the results of this study were consistent with those of previous studies. In this study, soil microbial diversity significantly decreased in herbicide contaminated soils. Although indigenous microbes could facilitate intrinsic remediation of chlorimuron-ethyl and atrazine contaminated soils, this process was particularly slow. Inoculation of strain CHL1 and ATR1 in contaminated soil effectively ameliorated the extent and duration of this inhibitory effect of chlorimuron-ethyl and atrazine.

The PCA analysis of all PLFAs showed that microbial community structure was significantly different between uninoculated and inoculated treatments. It was reported that incubation time, herbicide species and dose were the main factors affecting microbial community structure [51–53]. The results of this study were similar to those of previous reports. In this study, the microbial community structure changed substantially in relation to incubation time, herbicide and inoculation treatments. During the experiment, inoculation treatments had a significant effect on PLFA patterns, with significant variation in microbial community structure among all treatments on day 7, which was related to the detoxification of herbicides by indigenous microorganisms and inoculated strains of CHL1 and ATR1. Further, the inoculated degrading strains reduced the changes in soil microbial community structure, especially on day 7.

## Conclusion

In this study, strains CHL1 and ATR1 were combined to remediate chlorimuron-ethyl, atrazine and combined contaminated soils. The two herbicides alone and in combination generally decreased the concentrations of total PLFAs and the main microbial groups, decreased the Shannon-Wiener indices, and stimulated fungal PLFA concentrations. In addition, the microbial community composition was substantially altered in herbicide treatments. However, the inoculated treatments accelerated the degradation of chlorimuron-ethyl and atrazine in soils, especially in the combined treatment. Further, the effects of herbicides on soil microbial biomass, diversity and community structure were significantly reduced with inoculation. This study indicated that CHL1 and ATR1 have the potential to remediate chlorimuron-ethyl, atrazine and combined contaminated soils *in situ*.
